# Detection of Disease Features on Retinal OCT Scans Using RETFound

**DOI:** 10.3390/bioengineering11121186

**Published:** 2024-11-25

**Authors:** Katherine Du, Atharv Ramesh Nair, Stavan Shah, Adarsh Gadari, Sharat Chandra Vupparaboina, Sandeep Chandra Bollepalli, Shan Sutharahan, José-Alain Sahel, Soumya Jana, Jay Chhablani, Kiran Kumar Vupparaboina

**Affiliations:** 1 Department of Ophthalmology, University of Pittsburgh Medical Center, Pittsburgh, PA 15219, USA; stavshah11@gmail.com (S.S.); shv77@pitt.edu (S.C.V.); sab517@pitt.edu (S.C.B.); sahelja@upmc.edu (J.-A.S.); chhablanijk2@upmc.edu (J.C.); kkv@pitt.edu (K.K.V.); 2 Department of Electrical Engineering, Indian Institute of Technology Hyderabad, Hyderabad 502284, India; jana@ee.iith.ac.in; 3 Department of Computer Science, University of North Carolina at Greensboro, Greensboro, NC 27412, USA; a_gadari@uncg.edu (A.G.); s_suthah@uncg.edu (S.S.)

**Keywords:** retinal imaging, optical coherence tomography, machine learning, age-related macular degeneration, foundational model, automated report generation

## Abstract

Eye diseases such as age-related macular degeneration (AMD) are major causes of irreversible vision loss. Early and accurate detection of these diseases is essential for effective management. Optical coherence tomography (OCT) imaging provides clinicians with in vivo, cross-sectional views of the retina, enabling the identification of key pathological features. However, manual interpretation of OCT scans is labor-intensive and prone to variability, often leading to diagnostic inconsistencies. To address this, we leveraged the RETFound model, a foundation model pretrained on 1.6 million unlabeled retinal OCT images, to automate the classification of key disease signatures on OCT. We finetuned RETFound and compared its performance with the widely used ResNet-50 model, using single-task and multitask modes. The dataset included 1770 labeled B-scans with various disease features, including subretinal fluid (SRF), intraretinal fluid (IRF), drusen, and pigment epithelial detachment (PED). The performance was evaluated using accuracy and AUC-ROC values, which ranged across models from 0.75 to 0.77 and 0.75 to 0.80, respectively. RETFound models display comparable specificity and sensitivity to ResNet-50 models overall, making it also a promising tool for retinal disease diagnosis. These findings suggest that RETFound may offer improved diagnostic accuracy and interpretability for specific tasks, potentially aiding clinicians in more efficient and reliable OCT image analysis.

## 1. Introduction

**Figure 1:**
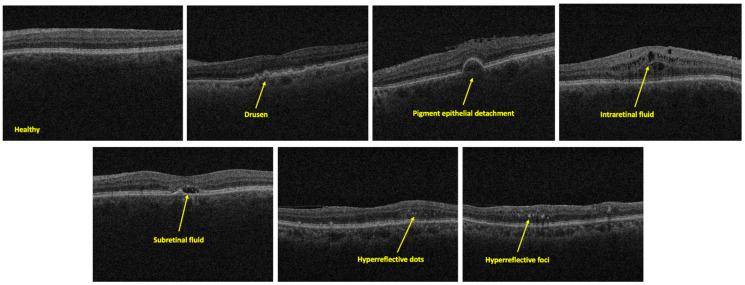
Examples of labeled OCT B-scans for drusen, pigment epithelial detachment, intraretinal fluid, subretinal fluid, hyperreflective dots, and hyperreflective foci.

Various eye diseases such as age-related macular degeneration (AMD) and diabetic retinopathy are the major causes of irreversible vision loss [1,2]. Accurate and early detection of these diseases is crucial in effective disease management. Optical coherence tomography (OCT) imaging enables clinicians with in vivo cross-sectional visualization of the posterior segment of the eye and the associated structural changes [3,4]. In particular, clinicians screen for the presence of specific pathologies, including subretinal fluid (SRF), intraretinal fluid (IRF), drusen, pigment epithelial detachment (PED), hyperreflective dots, and hyperreflective foci to diagnose the retinal disease (Figure 1). However, the current clinical practice of interpreting OCT images remains labor-intensive, subjective, and prone to variability [5]. Clinicians often encounter challenges in consistently assessing subtle pathological features and differentiating between healthy and diseased eyes, particularly when image quality is suboptimal [6,7]. The manual nature of OCT interpretation, combined with the increasing volume of scans in clinical settings, leads to diagnostic delays, inconsistencies, and potential misinterpretations, which can adversely affect patient care [8,9,10,11,12,13,14]. Against this backdrop, we aim to leverage artificial intelligence (AI) to automate the detection of key disease signatures on OCT [15,16,17,18].

The use of automated systems for analyzing OCT images has been an active area of research [5,19,20,21,22]. Traditional image-processing techniques have attempted to extract specific retinal layers and features like drusen and fluid using rule-based and machine learning methods. To identify retinal abnormalities, these approaches often relied on handcrafted features, such as intensity-based thresholding, texture analysis, and region-growing algorithms [23]. While these methods have shown promise, they tend to struggle with the variability in image quality and the complexity of retinal diseases, leading to inconsistent performance in real-world clinical applications. More recent efforts have focused on leveraging deep learning models to improve the accuracy and efficiency of retinal disease detection. Convolutional neural networks (CNNs) have been widely applied to OCT images for the classification of diseases such as AMD, diabetic macular edema (DME), and choroidal neovascularization (CNV) [24]. Studies using architectures like ResNet-50 [25,26], Inception-v3 [27], and VGG-16 [28] have demonstrated high accuracy rates in identifying key retinal biomarkers [29]. For example, ResNet-50 has been used extensively in medical imaging due to its ability to extract deep features, achieving an accuracy of 97.3% in classifying common retinal diseases from OCT scans [30]. A study by Leandro et al. using VGG-16 reported high accuracy (ranging from 93% to 99%) for detecting abnormalities like epiretinal membranes, intraretinal fluid, and macular neovascularization [31]. Despite their success, these CNN-based models are often limited by the need for large, annotated datasets, which are challenging to obtain in medical imaging.

Although deep learning models have demonstrated near-human-level performance on complex diagnostic tasks, acquiring and labeling the vast amounts of medical data required to train and test these models is both expensive and time-consuming. To address this challenge, foundation models have emerged, reducing the need to develop task-specific datasets from scratch [32,33,34,35]. These self-supervised transformer models are pretrained on diverse data, giving them broad applicability across multiple tasks [36,37,38,39,40,41]. Foundation models, which are well-established in natural language processing and computer vision, are now being applied to ophthalmic imaging to ease the burden of acquiring large, expertly labeled datasets. One such model is RETFound, a foundational model recently trained on 1.6 million unlabeled retinal OCT images [42]. RETFound captures interpretable features that can be used for various downstream tasks, making it a label-efficient tool for generalizing across retinal image analysis. Despite its potential, the application of RETFound to specific tasks, such as detecting pathological signatures in OCT scans, remains underexplored. By applying RETFound to identifying disease features in OCT images, we aim to enhance the model’s diagnostic value. Unlike many machine learning models, which often lack interpretability, RETFound offers the advantage of identifying key retinal signatures, providing clinicians with clearer insights into disease diagnosis. This approach improves interpretability compared to traditional convolutional neural networks (CNNs), offering a more intuitive understanding of disease-related biomarkers in retinal images.

In this study, we aim to evaluate the utility of RETFound to detect the presence/absence of key disease signatures in OCT B-scans. For those scans showing signs of disease, we further detect the presence of specific pathologies, including subretinal fluid (SRF), intraretinal fluid (IRF), drusen, pigment epithelial detachment (PED), hyperreflective dots, and hyperreflective foci. To this end, we built a custom annotation software and labeled a total of 1770 B-scans for disease features. Next, we trained the single task and multitask RETFound and ResNet-50 models on these scans and evaluated their performance.

## 2. Methods

### 2.1. Dataset

This retrospective study was conducted in accordance with the principles outlined in the Declaration of Helsinki, with approval from the institutional review board of the University of Pittsburgh Medical Center (UPMC), Pittsburgh. The approval number is Retrospective Study of Presentations & Outcome of Vitreo-Retinal Diseases (STUDY20030263). Informed consent was obtained as written consent from all participants for the inclusion of their retrospective data in the study. We utilized OCT volumes obtained from the Cirrus 5000 OCT device (Carl Zeiss Meditec, Jena, Germany) from 110 unique eyes corresponding to individual subjects diagnosed with age-related macular degeneration (AMD). Each Cirrus OCT volume comprised 128 B-scans, with a lateral resolution of 6 mm and a depth resolution of 2 mm, resulting in a pixel resolution of 512 × 1024. Approximately 16 B-scans were uniformly sampled from each volume, yielding a total of 1770 B-scans for analysis. Patients were screened between 2017 and 2019. These data were accessed on 31 May 2023 for research purposes, and authors did not have access to information that could identify individual participants during or after data collection.

### 2.2. Feature Description

The various AMD disease signatures labeled for training and testing the machine learning models include healthy or diseased scan, presence or absence of fovea, pigment epithelial detachment, drusen, subretinal fluid, intraretinal fluid, hyperreflective dots, and hyperreflective foci (Figure 1). Several of these features are also present in other retinal diseases such as diabetic retinopathy, central serous chorioretinopathy, and retinal vein occlusion.

### 2.3. Annotation Tool

**Figure 2:**
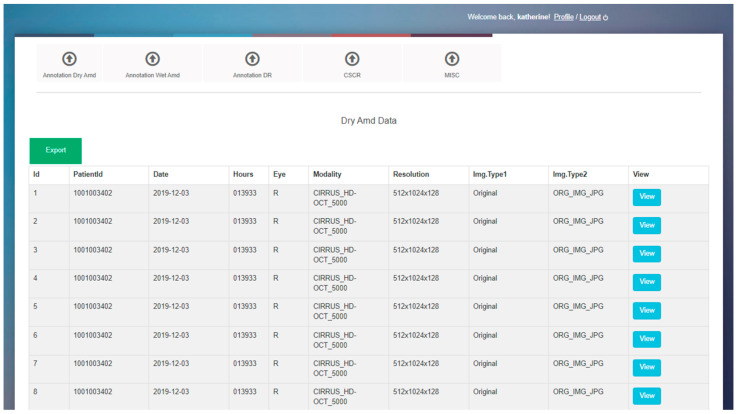
Main page of OCT image-labeling software created and used for labeling OCT scans.

**Figure 3:**
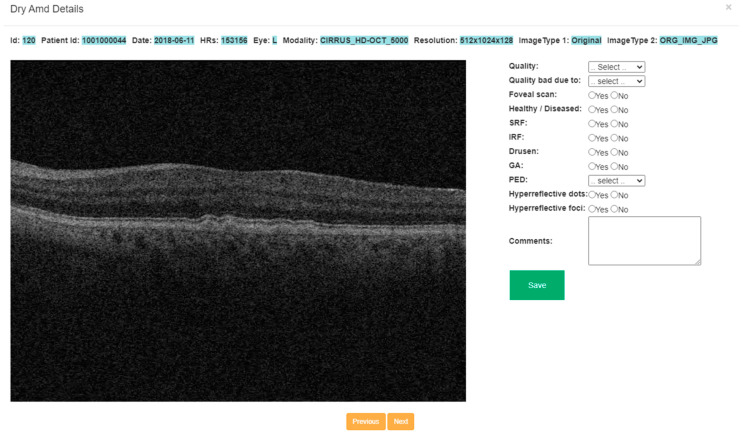
Interactive window pop-up of OCT scan used to label a single scan.

We developed an OCT image-labeling software in-house for annotating retinal features on scans (Figure 2). This software, created using PHP and SQL platforms, was deployed as a standalone application using an Apache server. The interface presents a table on the home screen listing each OCT scan along with its relevant characteristics, including patient research ID, laterality (right or left eye), imaging modality, resolution, and image type. There is a “View” button corresponding to each B-scan that users can select to perform the annotation. Clicking the “View” button takes the user to a different page where the user can view the full view of the B-scan with the list of features to annotate on the right of the image (Figure 3). There are check boxes for “Yes” or “No” for binary classification, i.e., for annotating the presence or absence of certain disease features. Further, there are also dropdown menus for some aspects of annotation, including type of PED. Accordingly, the user annotates each OCT image either by selecting options from the drop-down or by checking the check boxes for all aspects of the OCT image, including presence of specific retinal features and additional comments. Once an image is labeled by a user, the table updates the scan status from unlabeled to labeled. Annotations are saved, and users can proceed to the next OCT scan. The aggregated data of all labels can be downloaded for further analysis.

### 2.4. Annotation Strategy

One trained reviewer independently labeled the OCT B-scan features under consideration, with guidance from one retinal ophthalmologist. To ensure that the reviewer can label scan features in a standardized manner, the concurrence between this reviewer and the retinal ophthalmologist was established beforehand. Although this study only utilized one reviewer who labeled all the scans, the interrater reliability in labeling scan quality and pathological features was evaluated between this and another trained reviewer via concurrence analysis to determine potential discrepancies in labeling features [43]. Specifically, features including healthy/diseased scan, foveal scan, drusen, subretinal fluid, intraretinal fluid, and pigment epithelial detachment showed strong agreement (higher Cohen’s kappa coefficient), while hyperreflective dots and hyperreflective foci showed weaker agreement (lower Cohen’s kappa coefficient).

The one reviewer grades if the scan is a foveal scan or a non-foveal scan. Subsequently, the reviewer looks for the presence and absence of the disease features on OCT scans and grades them as healthy (scans with no retinal disease signatures) or diseased (scans with retinal disease signatures). If the scan is labeled as diseased, then the reviewer proceeds to annotate for the presence or absence of specific disease signatures including subretinal fluid, intraretinal fluid, drusen, PED, hyperreflective dots, and hyperreflective foci. Further, if PED was present, the type of PED was indicated as fibrovascular, flat irregular, serous, drusenoid, or hemorrhagic. The reviewer is provided with a “comments” box to make any specific comments about the image under consideration. This comprehensive annotation strategy aimed to capture the full spectrum of retinal characteristics and disease manifestations present in the OCT scans.

### 2.5. Data Preprocessing

Features represented in the downstream models include healthy/diseased scan, foveal scan, drusen, pigment epithelial detachment, and hyperreflective dots. The features of subretinal fluid, intraretinal fluid, and hyperreflective foci were represented sparsely in the training and testing datasets; hence, they were not included in the final models (Table 1).

### 2.6. RETFound Model

RETFound [42] is a foundational AI model trained on unlabeled OCT and color fundus photography (CFP) images in a self-supervised manner by using a masked autoencoder [44]. The image is divided into 16 × 16 patches of which 85% are randomly masked for OCT scans. The unmasked patches are flattened and fed into a large vision transformer encoder (ViT-large with 24 blocks and embedding vector size of 1024) to extract a latent vector representation of the image. This vector, along with masked tokens corresponding to the masked patches, are fed into a smaller Transformer decoder (ViT small with 8 blocks and an embedding vector size of 512) to produce reconstructed patches. The main aim of pretraining is to reproduce the original image with limited context (15% unmasked patches). This aids in learning meaningful latent representations which can be used for downstream tasks. The mean square loss between the reconstructed and original images is used as the loss function to train the model. Two separate models were trained on a large dataset of OCT (~730,000) and CFP (~900,000) images. These models performed effectively on many downstream tasks involving ocular disease diagnosis, prognosis, and systemic disease prediction. The main advantage of such a large foundational image model trained in a self-supervised manner is that it eliminates the need for a large, labeled dataset by learning meaningful representations of the images. 

**Figure 4:**
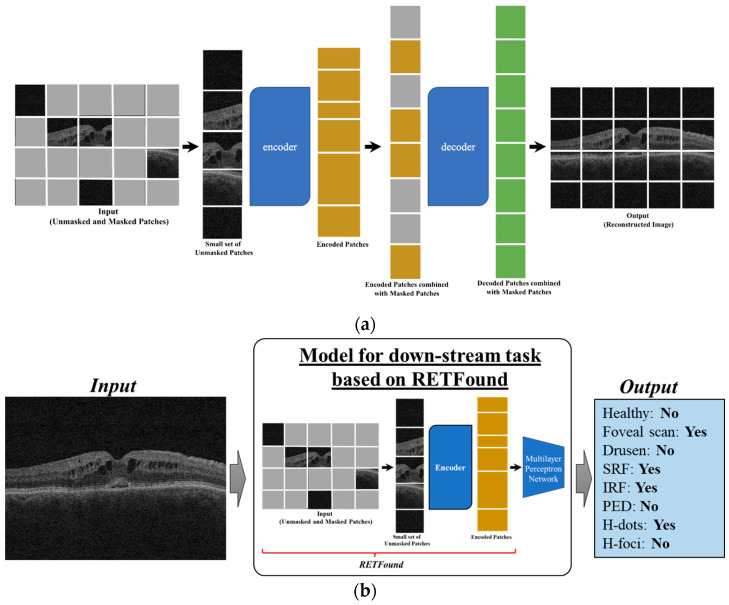
(**a**) Schematic of RETFound model and (**b**) model for down-stream task of labeling pathologic features on OCT scans based on RETFound.

In this work, we used the RETFound encoder [42,44] (Figure 4a) and added a multilayer perceptron (MLP) network for classification (Figure 4b). The images were resized to 224 × 224 and duplicated to produce 3 channels to ensure compatibility with the model’s input requirements. Apart from this, standard transformations like color jitter and normalization were also implemented. We then finetuned the model with our dataset using an Adam optimizer with a learning rate of 1 × 10^−4^. The model was trained for 25 epochs, and fine-tuning involved changing all the parameters of the model, including the encoder to minimize the Binary Cross Entropy Loss.

We specifically compared two variants of the RETFound model, a multitask classifier and multiple single-task classifiers. The multitask classifier approach uses a common backbone and an additional linear layer with a five-dimensional (corresponding to the number of features) output layer and sigmoid non-linearity. Each value corresponds to the probability of the presence of a particular feature. For the multiple single-task classifiers approach, we trained completely independent models for each feature. A sigmoid non-linearity was used to produce a scalar value corresponding to the probability of presence of the feature.

### 2.7. ResNet-50 Model

**Figure 5:**
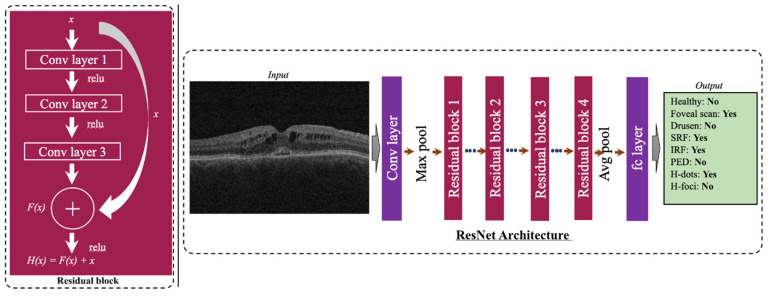
Schematic of the ResNet-50 model trained on the task of labeling pathologic features on OCT scans.

To check the efficacy of the self-supervised RETFound model, we compared it with a ResNet-50 [25] baseline (Figure 5). A similar set of transformations as mentioned for the RETFound model was conducted before feeding the images to the ResNet-50 model. The ResNet-50 pretrained on ImageNet [45] was used to initialize the weights. The same multilayer perceptron layers are added to the ResNet-50 backbone. The model is finetuned with our dataset using an Adam optimizer with a learning rate of 1 × 10^−4^. The model was trained for 25 epochs, and fine-tuning involved minimizing the Binary Cross Entropy Loss. We compared two variants of the ResNet-50 model, a multitask classifier and multiple single-task classifiers, with the same architecture as the RETFound model, respectively.

## 3. Results

We compared the RETFound model with ResNet-50 pre-trained on ImageNet [45] for both multiple single-task classifiers and a multitask classifier. The models were trained to identify healthy or diseased scans, the presence or absence of fovea, drusen, pigment epithelial detachment, and hyperreflective dots.

Table 2 shows the evaluation metrics for each feature in the ResNet-50 and RETFound models. Overall, the AUC-ROC values are similar for all model types, ranging from 0.75 to 0.80. AUC-ROC is a suitable evaluation metric for imbalanced datasets to understand how a model performs across different thresholds. Since many pathological features in our dataset have classes that are not represented equally, we can gauge model performance using AUC-ROC. Hence, the excellent AUC-ROC values signify that both ResNet-50 and RETFound models are effective at differentiating between the positive and negative classes. More specifically, the average AUC-ROC is highest for the RETFound model trained on individual classifiers (0.80), followed by the ResNet-50 single-task architecture (0.78), ResNet-50 multitask architecture (0.76), and RETFound multitask architecture (0.75).

The accuracy of diagnosing healthy versus diseased OCT B-scans is high for all model types (ResNet-50 single task = 0.77, RETFound single task = 0.76, ResNet-50 multitask = 0.78, RETFound multitask = 0.74), as shown in Table 2. For specific features, the accuracies in identifying foveal scan and PED indicate effective model performance, while drusen and hyperreflective dots show lower accuracies. On average, specificity is higher for the RETFound single task and multitask models, while sensitivity is higher for the RETFound single task and ResNet-50 multitask models.

For external validation of our ResNet-50 and RETFound models, we utilized the OCT dataset from Kermany et al. (2018) [46]. We used the test set consisting of 968 images from Heidelberg Spectralis OCT devices that aligned with our finetuned features, specifically healthy/diseased and drusen. We used our single-task models for the two separate tasks: predicting healthy/diseased and detecting presence/absence of drusen. Similar pre-training as with our internal dataset was performed on these OCT scans. As shown in Table 3, for both ResNet-50 and RETFound models, the accuracies are high for predicting healthy/diseased and drusen (0.94 for all). Sensitivity is slightly yet statistically significantly higher for RETFound in both features, while specificity is the same for both models. The AUC-ROC is almost perfect, 0.99 and 0.98, respectively, for healthy/diseased and drusen in RETFound and 0.98 for both features of ResNet-50 (Table 3).

To compare our models in low-shot learning conditions, we randomly extracted 30 scans each from our internal dataset and external (Kermany) dataset and used the ResNet-50 and RETFound models for feature classification. The results, presented in Table 4, show that for the internal dataset the ResNet-50 and RETFound models show higher evaluation metrics for different features in the single-task model, while RETFound generally performs better in the multitask model. For the external dataset, RETFound shows higher accuracy, sensitivity, specificity, and AUC-ROC for both healthy/diseased classification and drusen identification, with drusen showing a statistically significant difference between the proportions. Additionally, there is a statistically significant difference in the AUC-ROC values of the external dataset but not the internal dataset.

## 4. Discussion

This study shows that the RETFound model performs similarly well to ResNet-50 on the specific task of disease feature detection on retinal OCT scans. The accuracy and AUC-ROC values of the RETFound model in detecting diseased scans, foveal scans, drusen, PED, and hyperreflective dots is comparable to the ResNet-50 model, for both single-task and multitask architectures. As RETFound is a foundational model that learns generalizable representations from unlabeled retinal images, it has the advantage of increased interpretability compared to the ResNet-50 model, which may aid ophthalmologists by providing more reliable representations for automated disease detection. Additionally, RETFound uses self-supervised learning, which decreases the demand for experts to undergo the effortful and time-consuming process of labeling data for model training and evaluation.

Hence, RETFound may be a suitable model architecture for machine learning models in ophthalmology, to create decision-support algorithms for detecting disease pathology. These automated algorithms can help standardize medical diagnoses and increase the accessibility of OCT diagnostic tools. The accuracies of the model in detecting specific disease features also give clinicians an idea into which features have stronger diagnostic capacity and may be more reliable, such as drusen and PED, compared to hyperreflective dots. As evidenced by our previous concurrence analysis, a lower Cohen’s kappa coefficient was observed when reviewers annotated hyperreflective dots and hyperreflective foci [43]; hence, the limited ability of the machine learning models to discern hyperreflective dots may be a combination of subjective labels from reviewers and difficulties discerning hyperreflective material on scans by the machine learning models. On the other hand, the reviewers previously demonstrated strong concurrence in labeling other pathological features, including healthy/diseased scan, foveal scan, drusen, and PED. The RETFound and ResNet-50 models were able to process these features with stronger accuracy, likely partly due to better quality labels.

Compared to our dataset, the Kermany et al. (2018) [46] dataset used for external validation demonstrated higher evaluation metrics, with accuracy, sensitivity, specificity, and AUC-ROC all exceeding 0.90 for both ResNet-50 and RETFound models. Notably, in low-shot learning scenarios, RETFound consistently showed higher evaluation metrics on both internal and external datasets, particularly for drusen classification. This indicates that the model is robust, generalizable, and reliable across varied patient populations and imaging devices. The discrepancy in evaluation metrics may be attributed to differences in imaging equipment: our dataset comprises OCT scans from the Cirrus 5000 OCT (Carl Zeiss Meditec), while the external dataset includes scans from the Spectralis OCT (Heidelberg Engineering), known for its higher image resolution. Consequently, models may perform better on Spectralis images due to improved quality and clearer visual representation of features, such as larger drusen and more pronounced pathology. Nonetheless, evaluating our model on Cirrus OCT scans remains crucial, as they are widely used in many clinical settings globally.

Limitations of this study include the inability to evaluate the RETFound and ResNet-50 models on detecting subretinal and intraretinal fluid, hyperreflective foci, and type of PED due to the lack of a substantial quantity of labeled scans presenting these features. Additionally, as all scans were from the Cirrus 5000 OCT device (Carl Zeiss Meditec) in patients with AMD, the capability of these models to capture pathological features of other retinal diseases and in other OCT devices are not examined. Lastly, OCT B-scan quality is an important determinant of the diagnostic accuracies for machine learning models [6,47,48,49]; hence, the classification of scan quality into bad, usable, and good scans should be explored in future work.

This study shows that the generalizable representations created by the RETFound model are suitable for a specific pathologic feature classification task using retinal OCT scans. RETFound performs with about the same accuracy and AUC-ROC as the ResNet-50 models and may even perform better on classifying certain disease pathologies, such as drusen. Additionally, RETFound requires less labeled data and is more interpretable compared to ResNet-50. The widespread use of electronic medical records with ophthalmic imaging allows for the integration of artificial intelligence image analysis tools for computer-aided diagnosis. RETFound models may be uniquely suitable in performing certain ophthalmic disease classification tasks. The conclusions from this analysis can be implemented in engineering decisions while creating AI models for retinal disease diagnosis and tracking progression over time.

## Figures and Tables

**Table 1:** Counts of each feature represented in the training and testing sets. Due to low counts for subretinal fluid, intraretinal fluid, and hyperreflective foci, these features were not included in model training and evaluation.

	Training Set (1360 Scans)	Testing Set (410 Scans)
Healthy scan	420	165
Diseased scan	940	245
Foveal scan	135	33
Subretinal fluid	89	2
Intraretinal fluid	69	6
Drusen	227	91
Pigment epithelial detachment	220	27
Hyperreflective dots	840	257
Hyperreflective foci	40	32

**Table 2:** Evaluation metrics for single-task and multitask ResNet-50 and RETFound models on retinal scan characteristics and disease signatures for our internal dataset. Notation: RN50—ResNet-50; RF—RETFound; H/D—healthy/diseased; H-Dots—hyperreflective dots. Higher values between RN50 and RF are bolded; statistically significant differences (using two-sample z-test for proportions) are represented with asterisks.

	Evaluation Metrics							
	Accuracy		Sensitivity		Specificity		AUC-ROC	
**Single Task**	***RN50***	***RF***	***RN50***	***RF***	***RN50***	***RF***	***RN50***	***RF***
H/D	**0.77**	0.76	**0.71**	0.62	0.81	**0.86**	**0.83**	0.80
Foveal scan	**0.94**	**0.94**	0.88	**0.94**	**0.95**	0.93	0.94	**0.95**
Drusen	0.69 *	0.76 *	0.65 *	0.78 *	0.70	**0.75**	0.75	**0.83**
PED	0.84 *	0.79*	0.44 *	0.63 *	**0.86**	0.81	0.74	**0.76**
H-Dots	**0.62**	**0.62**	0.82 *	0.67 *	0.28 *	0.54 *	0.64	**0.66**
Average	**0.77**	**0.77**	0.70	**0.73**	0.72	**0.78**	0.78	**0.80**
**Multitask**	***RN50***	***RF***	***RN50***	***RF***	***RN50***	***RF***	***RN50***	***RF***
H/D	0.78 *	0.74 *	0.66	**0.72**	0.87 *	0.76 *	0.80	**0.81**
Foveal scan	0.93	**0.94**	0.94 *	0.85 *	0.93	**0.94**	**0.96**	0.91
Drusen	0.70 *	0.60 *	0.66 *	0.44 *	**0.72**	0.65	0.74 *	0.60 *
PED	**0.82**	0.81	0.37	**0.48**	**0.84**	**0.84**	0.69	**0.74**
H-Dots	0.62	**0.64**	**0.80**	0.72	0.30 *	0.50 *	0.65	**0.67**
Average	**0.77**	0.75	**0.69**	0.64	0.73	**0.74**	**0.76**	0.75

**Table 3:** Evaluation metrics for external validation on independent dataset from Kermany et al. (2018) [46]. Notation: RN50—ResNet-50; RF—RETFound; H/D—healthy/diseased. Higher values between RN50 and RF are bolded; statistically significant differences (using two-sample z-test for proportions) are represented with asterisks.

	Evaluation Metrics							
	Accuracy		Sensitivity		Specificity		AUC-ROC	
**Single Task**	***RN50***	***RF***	***RN50***	***RF***	***RN50***	***RF***	***RN50***	***RF***
H/D	**0.94**	**0.94**	0.90 *	0.92 *	**0.95**	**0.95**	0.98	**0.99**
Drusen	**0.94**	**0.94**	0.91 *	0.92 *	**0.95**	**0.95**	**0.98**	**0.98**

**Table 4:** Evaluation metrics for validation on internal dataset and external dataset from Kermany et al. (2018) [46]. Notation: RN50—ResNet-50; RF—RETFound; H/D—healthy/diseased. Higher values between RN50 and RF are bolded; statistically significant differences (two-proportion z-test for accuracy, sensitivity, specificity) are represented with asterisks. Two-sample z-test for proportions on AUC-ROC shows no statistically significant difference for the single-task and multitask internal dataset and a statistically significant difference for the external dataset.

	Evaluation Metrics							
	Accuracy		Sensitivity		Specificity		AUC-ROC	
*Internal Dataset*								
**Single task**	***RN50***	***RF***	***RN50***	***RF***	***RN50***	***RF***	***RN50***	***RF***
H/D	**0.69**	0.57	**0.61**	0.58	0.74 *	0.56 *	0.75 *	0.59 *
Foveal scan	**0.74**	0.70	0.82 *	0.97 *	**0.73**	0.68	0.79 *	0.91 *
Drusen	0.59	**0.69**	**0.57**	0.56	0.60 *	0.72 *	0.56	**0.66**
PED	0.81 *	0.50 *	0.26 *	0.78 *	0.85 *	0.48 *	**0.64**	0.63
H-Dots	0.51 *	0.68 *	0.36 *	0.71 *	0.75 *	0.62 *	0.54 *	0.70 *
Average	0.52	**0.61**	0.63	**0.66**	0.67	**0.70**	0.72	**0.73**
**Multitask**	***RN50***	***RF***	***RN50***	***RF***	***RN50***	***RF***	***RN50***	***RF***
H/D	**0.64**	0.55	0.16 *	0.67 *	0.96 *	0.51 *	0.70	**0.74**
Foveal scan	0.53 *	0.95 *	0.91	**0.94**	0.50 *	0.95 *	0.75 *	0.96 *
Drusen	0.52	**0.55**	0.32 *	0.67 *	0.96 *	0.51 *	**0.70**	0.64
PED	0.70	**0.72**	0.44 *	0.67 *	0.72	**0.73**	0.58	**0.71**
H-Dots	0.54	**0.63**	0.49 *	0.67 *	**0.63**	0.56	0.60	**0.69**
Average	0.61	**0.68**	0.46 *	0.73 *	0.75 *	0.64 *	0.67	**0.75**
*External (Kermany) Dataset*								
**Single task**	***RN50***	***RF***	***RN50***	***RF***	***RN50***	***RF***	***RN50***	***RF***
H/D	0.84	**0.89**	0.82	**0.91**	0.81	**0.88**	0.92	**0.95**
Drusen	0.50 *	0.93 *	0.38 *	0.92 *	0.53 *	0.94 *	0.44 *	0.84 *

## Data Availability

Data cannot be shared publicly as they contain potentially sensitive patient information. However, data may be available to researchers who meet the criteria for access to confidential data, pending approval from the University of Pittsburgh Medical Center’s institutional ethics committee. Interested researchers can request access by contacting the corresponding author at kiran1559@gmail.com.
